# Data Fusion of RGB and Depth Data with Image Enhancement

**DOI:** 10.3390/jimaging10030073

**Published:** 2024-03-21

**Authors:** Lennard Wunsch, Christian Görner Tenorio, Katharina Anding, Andrei Golomoz, Gunther Notni

**Affiliations:** 1 Group of Quality Assurance and Industrial Image Processing, Faculty of Mechanical Engineering, Technische Universität Ilmenau, 98693 Ilmenau, Germany; christian.goerner-tenorio@tum.de (C.G.T.); katharina.anding@idmt.fraunhofer.de (K.A.); andrei.golomoz@tu-ilmenau.de (A.G.); gunther.notni@tu-ilmenau.de (G.N.); 2 Fraunhofer Institute for Applied Optics and Precision Engineering IOF Jena, 07745 Jena, Germany

**Keywords:** multimodal imaging, data fusion, registration, RGB-D, joint bilateral upsampling, Markov random fields, data enhancement

## Abstract

Since 3D sensors became popular, imaged depth data are easier to obtain in the consumer sector. In applications such as defect localization on industrial objects or mass/volume estimation, precise depth data is important and, thus, benefits from the usage of multiple information sources. However, a combination of RGB images and depth images can not only improve our understanding of objects, capacitating one to gain more information about objects but also enhance data quality. Combining different camera systems using data fusion can enable higher quality data since disadvantages can be compensated. Data fusion itself consists of data preparation and data registration. A challenge in data fusion is the different resolutions of sensors. Therefore, up- and downsampling algorithms are needed. This paper compares multiple up- and downsampling methods, such as different direct interpolation methods, joint bilateral upsampling (JBU), and Markov random fields (MRFs), in terms of their potential to create RGB-D images and improve the quality of depth information. In contrast to the literature in which imaging systems are adjusted to acquire the data of the same section simultaneously, the laboratory setup in this study was based on conveyor-based optical sorting processes, and therefore, the data were acquired at different time periods and different spatial locations. Data assignment and data cropping were necessary. In order to evaluate the results, root mean square error (RMSE), signal-to-noise ratio (SNR), correlation (CORR), universal quality index (UQI), and the contour offset are monitored. With JBU outperforming the other upsampling methods, achieving a meanRMSE = 25.22, mean SNR = 32.80, mean CORR = 0.99, and mean UQI = 0.97.

## 1. Introduction

Computer vision using artificial intelligence is being integrated into many industrial processes to improve their performance by supervising the processes, collecting information for defect reduction [1,2], object localization [3], and obtaining more knowledge about the process. With better access to depth data and its addition to pre-installed computer vision systems, the range of applications of computer vision within quality insurance and decision-making has increased. In applications such as bridge inspection [1], railway quality insurance [2], agriculture [3,4], and robotics [5], RGB-D data are being utilized. By adding RGB data to depth data, the collection of shape-based information in addition to texture- and color-based information RGB-D data is generated. These data can ensure a deeper understanding of industrial processes and their defect sources. Therefore, applications such as approximate weight determination, defect size determination, defect localization, and additional support in decision-making can benefit from RGB-D data.

In order to create RGB-D data from a multi-view system, a data fusion algorithm is necessary to combine RGB and depth data. By fusing data, information from different sources are combined into one data object. However, a combination of these data can be challenging since different information sources can have different attributes. Data fusion can be beneficial not only in terms of additional information but also for an improvement in data quality, as shown, for example, by Okafor et al. [6] and Nemati et al. [7]. Given the capabilities of different systems, a smart combination of these systems can result in the compensation of the disadvantages of each system respectively. In order to process images with different resolutions and boundaries, more complex methods are needed. Boström et al. [8] collected multiple definitions of information fusion. The goals of information fusion are defined as “the provision of a better understanding of a given scene” and to “obtain information of greater quality”, among others. That is why RGB-D data have such vast potential when implemented in industrial processes for quality insurance.

Setting up a high-resolution sensor system for RGB-D data imaging can be expensive since at least two cameras are necessary, with one of them imaging the depth data while the other images RGB data, depending on the imaging techniques, and costs can become high. In order to obtain the precise RGB-D data from an analyzed sample, the sensor systems have to be either spatially adjusted, e.g., inclined under a certain angle towards each other to capture the same section of the sample at the same time, or temporally adjusted, e.g., not capturing the same sample section and adjusting the time shift due to different spatial acquisition positions via object tracking. The adjustment of the sensor systems is carried out via a co-ordinate system transformation of one type of data into the co-ordinate system of another system. Regarding sensor systems, there are many different setups for color- and shape-based imaging. Siepmann et al. [9] used a setup that utilized pattern projection. Another method with which to obtain RGB-D images is the combination of known depth imaging systems, such as the time of flight (ToF) [10,11,12,13], confocal microscopy [14], light detection and ranging (LIDAR) [15], or laser scanning [16,17,18] in combination with an RGB camera system.

Hach et al. [19] present an RGB-D camera containing a combination of a ToF sensor and an RGB camera. Both systems are combined in the system without inclination to each other. A typical system used in the literature [20,21,22] for a combination of a ToF sensor and an RGB camera is the Microsoft Kinect system. Another possibility for generating multimodal data is the combination of thermal data acquired by thermal infrared (TIR) cameras and depth imaging systems [16,17,23,24,25]. Regarding the adjustment of the sensor systems, different acquisition structures can be differentiated. These structures are either stationary, e.g., fixing the different imaging systems onto a stationary structure [12,13,15,16,17], or dynamic structures, e.g., a terrestrial scanner moving in the ambient air [15,18,20,23,26]. In order to summarize the state-of-the-art multimodal imaging for obtaining depth and color information, different depth-imaging systems are viable depending on the specific application.

Setting up an RGB-D imaging system can also become quite complex [27,28]. The more the camera parameters are the same, such as resolution, the easier it is to implement the data fusion algorithm. However, high-resolution depth cameras are more expensive than RGB cameras of the same resolution. Adjusting both cameras is also a challenging but necessary task. By calibrating both systems, the imaged regions are able to overlap and, thus, enable more precise data fusion. Therefore, a lot of thought needs to be put into setting up a viable imaging system for fused RGB-D data before data processing.

This work will analyze the required samples with a laboratory setup based on optical sorting processes [29,30]. Industrial processes based on optical sorting using a conveyor belt need to analyze a high quantity of samples in a short period of time. Therefore, only two depth acquisition methods, ToF and laser triangulation, respectively, should be considered for laser scanning in more detail. Other methods, such as interferometry, confocal microscopy, pattern projection, or photogrammetry, have a higher resolution than ToF or laser scanning methods but require more measuring time and come with greater costs. Due to the higher spatial resolution of a non-time-based measurement compared to ToF, this work uses a triangulation-based laser line scanning system as an effective depth imaging system. After setting up the imaging system, a fitting algorithm was needed for data fusion. In the past, a wide range of such data fusion methods have been proposed. Eichhardt et al. [11] give an overview of data fusion methods. A brief overview of different data fusion classifications, techniques, and architectures is given by Castanedo [31] and Elmenreich [32].

The fitting algorithm, also called data fusion, consists of data acquisition and preprocessing in case of different camera resolutions, as well as up or downsampling and the co-ordinate system transformation of one data type into the co-ordinate system of the reference system [33]. Usually, the system with the higher resolution is the preferred reference system; however, due to hardware limitations, this is not always viable. When transforming one image into the co-ordinate system of the reference system, false values can occur. These are pixel values that are seen by one system but not the other. The way these false values are dealt with is managed by the upsampling algorithm used to adjust the image resolution before transforming the co-ordinate system [34]. Therefore, this paper focuses on the variation of upsampling algorithms.

In the following sections, first, the data and imaging setup used in this work are introduced, and some background on data preprocessing is given; then, different upsampling methods, such as joint bilateral upsampling (JBU) [35,36] and Markov random fields (MRFs) [37,38], and different interpolation algorithms [39,40] are briefly described. For further reading on the matter of data fusion, Junger et al. [41] also compare multiple data fusion methods. In Section 4, the complete process of data acquisition, preprocessing, and data fusion, including post-processing, is described in detail. Afterward, a closer look at the evaluation process and its parameters is given. Finally, the results of each data fusion method applied to the same raw images are shown and evaluated based on the root mean square error (RSME), correlation (CORR), signal-to-noise ratio (SNR), universal quality index (UQI), and contour offset ($\Delta {\mathrm{Off}}_{position}$).

## 2. Data Fusion Process

Data fusion is the process of combining data from different sources [42]. By doing so, one can gain deeper knowledge and access combined information. The resolution, offset, and regions of interest (ROI) of the images must be adjusted by the data fusion algorithm so that each pixel of the RGB image gets a corresponding depth value in addition to the color values. This progress is difficult due to an offset in images both in the translational and rotational direction caused by different resolutions, non-commensurability, and missing or inconsistent data, as described by Illmann et al. [43] and Lahat et al. [44].

The main steps of data fusion are the following [33]:

- Data acquisition and cropping;
- Feature extraction;
- Data registration;
- Post-processing.

The ROI is defined by a cropping algorithm that crops the objects and works as a segmentation algorithm. In order to adjust the resolution of the data, the up- or downsampling and the combination of both must be realized. For this process, a reference image must be established before the resolution of the other image is adjusted. In this paper, the RGB image was chosen as the reference image for the fused RGB-D image. In addition to the higher resolution, it contains fewer defects and contains sharper imaged edges. The offset can be dealt with by translating the depth image based on information from an object registration algorithm. In order to accomplish this, the image-based data must be transformed into the same co-ordinate system to represent correlating areas. For this data registration, extracting the corresponding features in each image is necessary. Illmann et al. [43] provide further details and strategies for merging data. Feature extraction and data registration also deal with the time delay between both imaging systems. Therefore, both offset and time delay are solved.

This paper compares three simple interpolation methods with more complex methods, such as MRF and JBU, to achieve RGB-D-fused data. In the following, each upsampling method is presented in more detail.

### 2.1. Interpolation Methods

The classic interpolation methods used in this paper are given by Dianyuan [39] and Nischwitz et al. [40]. These methods were used as direct fusion methods to obtain corresponding depth pixel values for each respective RGB pixel. First, nearest-neighbor interpolation is chosen, where the unknown value of a pixel is calculated based on the interpolation of the pixel closest to the new pixel position. The second interpolation method used is a bi-linear interpolation, where four weight pixels are used for value calculation. The third and last interpolation method uses a cubic interpolation. Here, the unknown pixel is calculated using 16 surrounding pixels. Moreover, the interpolation methods are basic methods in image processing and are used in MRF and JBU as well.

### 2.2. Joint Bi-lateral Upsampling

Joint bi-lateral upsampling [35] is a guided depth upsampling method that uses bi-lateral filters to calculate a high-resolution depth map, $D^{h}$. It is considered a local depth upsampling method [11] because the weight for the convolution is calculated based on the local pixel position. JBU uses the weighted convolution of a high-resolution RGB image, $I^{h}$, with a lower-resolution depth map, $D^{l}$. The weight can be calculated with a range filter, *r*, which is a Gaussian kernel with $\sigma_{r}=0.5$, and the intensity differences of the high-resolution image, $I^{h}$, and the low-resolution image, $I^{l}$, at their pixel positions. The high-resolution depth map can be obtained with the simplified Equation (1) [45]:

(1) $$D_{p}^{h}=\frac{\sum_{q\in S}w\left(p,q\right)\;\cdot \;D_{q}^{l}}{\sum_{q\in S}w\left(p,q\right)},\;\mathrm{with}\;\;w\left(p,q\right)=r\;\cdot \;\left(I_{p}^{h}-I_{q}^{l}\right)$$

where *S* is the neighborhood of the pixel, *p* is the pixel position of the high-resolution data, and *q* is the pixel position of the low-resolution data. Since JBU is a guided upsampling method, to apply this method, the resolution of both data needs to be an integer multiple of each other. In order to apply JBU, the resolution difference between $D_{p}^{h}$ and $D_{q}^{l}$ needs to be an integer multiple of two. Moreover, Riemens et al. [45] proposed a multistep upsampling method to smoothen the edges. The multistep upsampling approach utilizes a cascade of 2×2 upsampling factors to reach factors of 4×4 or 8×8.

### 2.3. Markov Random Fields

Depth upsampling by Markov random fields (MRFs) [37,38] is a guided, global method [11] because it is used as an optimization method to find the global optimum. It utilizes a Markov network to calculate the pixel values of the upsampled depth map based on weighted high-resolution image data and estimated upsampled depth data. With an iterative process, the current calculated depth map can be optimized by considering the high-resolution image data. Liu et al. [46] proposed a model that considers a data term and a smoothing term to calculate the high-resolution depth map. The data term calculates the quadratic differences between the real and estimated data, and the smoothing term calculates the given potential with a weight that depends on the given image data. Equation (2) [46] shows the proposed model:

(2) $$D^{h}=argmin_{D}\left\{\left(1-\beta \right)\;\cdot \;\sum_{i\in p}{\left(D_{i}-D_{i}^{0}\right)}^{2}+\beta \;\cdot \;\sum_{i\in p}\sum_{j\in S}w_{i,j}^{c}{\left(D_{i}-D_{j}\right)}^{2}\right\},$$

where β is an empirical factor, *i* and *j* are indices, and $D^{0}$ is the initial cubic interpolated high-resolution depth map of the low-resolution depth map. The weight is given in Equation (3) [46]:

(3) $$w_{i,j}^{c}=exp\left(-\frac{\sum_{k\in C}|I_{l}^{k}-I_{j}^{k}{|}^{2}}{6\;\cdot \;\sigma_{c}^{2}}\right),$$

where *C* represents the three RGB values, *k* is an index, and $\sigma_{c}$ is an empirical factor. Deriving Equation (2) [46] and setting it equal to zero leads to Equation (4) [46]:

(4) $$D_{i}^{n+1}=\frac{\left(1-\beta \right)\;\cdot \;D_{i}^{0}+2\;\cdot \;\beta \;\cdot \;\sum_{j\in S}w_{i,j}^{c}\;\cdot \;D_{j}^{n}}{\left(1-\beta \right)+2\;\cdot \;\beta \;\cdot \;\sum_{i\in S}w_{i,j}^{c}},$$

where *n* corresponds to the current iteration.

## 3. Hardware and Data Preprocessing

Industrial processes are usually stationary systems; therefore, the lab setup used for this work is also a stationary system. Since the integration of an imaging system into an industrial process can be expensive and complex, a conveyor belt system was chosen. These systems are widely utilized in industry and provide simpler setup options compared to free-falling objects. However, one should note that when imaging the depth information of free-falling objects, the complete object can be imaged when using conveyor belts, but the bottom of the object cannot be imaged due to the top-down view of the system [47]. Therefore, only a 2.5-dimensional image can be generated.

In order to image objects in the same scene in an industrial environment, a setup consisting of a feeding system and an imaging system was built. In the feeding system, two conveyor belts are moved at different speeds, and five plates are arranged horizontally along the second belt to separate the incoming objects. The imaging system is positioned along the second belt. This system consists of an RGB line camera and a 3D laser line scanner. As described in Section 1, for depth imaging, a 3D laser line scanner was chosen since, when compared to systems such as the time of flight system of Microsoft Kinect, it obtains a better overall resolution (see Refs. [48,49]). The 3D laser line scanner images a 3D point cloud. However, JBU, MRFs, and interpolation upsampling were proposed for the depth map data. Therefore, the imaged point cloud was transferred into a depth map.

Both cameras were chosen based on each other’s parameters to ease the data fusion process later on. Although imaging objects placed on a conveyor belt results in a shadow below the object due to illumination, this is a cheaper alternative. The shadow below the object does not exist with planar objects and is not relevant for the detection of defects on the surface of the object. The important attributes of each camera are shown in Table 1 [49,50]. The number of active pixels for the laser line scanner was reduced by binning due to the necessary speed of the converter belt to ensure the proper separation of the objects. Using the full scanner would have resulted in missing pixels in the depth image.

Since the cameras optical axes are horizontally shifted, the number of pixels covering the ROI (the conveyor belt) is not the same as the number of active pixels. According to the definition of ROI, both sensor systems lose some pixels at the edges. Therefore, the ratio between the used pixels of both systems is no longer exactly two; it is a decimal number. This is important for the use of JBU and will be explained in Section 2.2.

**Figure 1:**
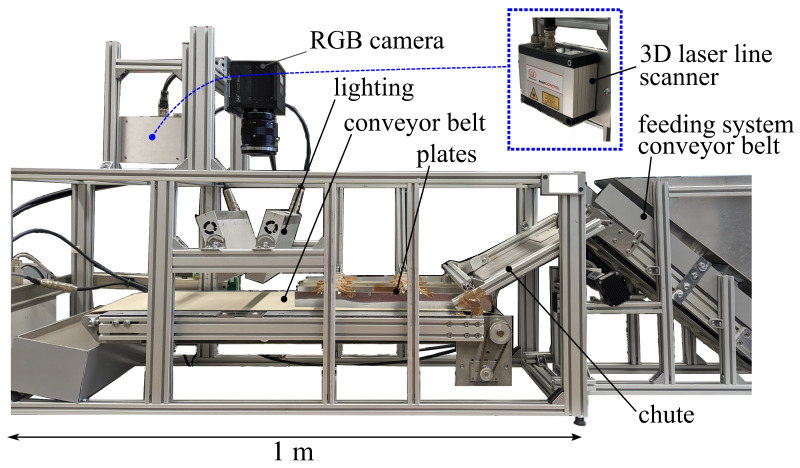
Setup used for 3D and RGB imaging.

The laboratory setup by Anding et al. [29,30] is shown in Figure 1. The feeding system with object separation can be seen on the right, and the imaging area is next to the separation plates. Both cameras are positioned above the illumination system and imaging area. The separation of objects is implemented by the combination of two conveyor belts moving at different speeds and the array of separation plates, which are arranged orthogonal to the movement direction of the second conveyor belt. In order to accomplish homogeneous illumination for the reduction of object shadows, the objects were illuminated using two angled light sources from above and one light source below the conveyor belt. For depth data acquisition, no additional illumination was needed.

This study features natural objects such as stones from quarries. These complex objects were chosen to show the limits of the presented data fusion approach. Since they don’t have a planar surface, the missing depth data below the objects need to be considered. A coin was used as a simple known round object for the validation of each individual process. In order to avoid losing pixels while imaging, the line frequency is calculated automatically. In this way, the speed of the conveyor belt in the imaging path and the line frequency of both cameras can be adjusted. An incorrect line frequency results in compressed or stretched images. A round normal object and Equation (5) were used to find the correct line frequency, since the round normal object is imaged as an ellipse at incorrect line frequencies:

(5) $$\frac{x^{2}}{a^{2}}+\frac{y^{2}}{b^{2}}=1$$

**Figure 2:**
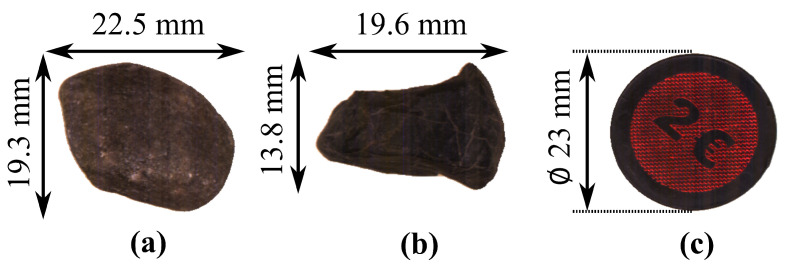
Example objects utilized: (**a**) stone-A, (**b**) stone-B, and the (**c**) validation coin.

With *a* and *b* describing both ellipsoid axles. These parameters are calculated using elliptical Hough transformation. Figure 2 shows examples of the objects of the featured dataset and the coin used for validation.

**Figure 3:**
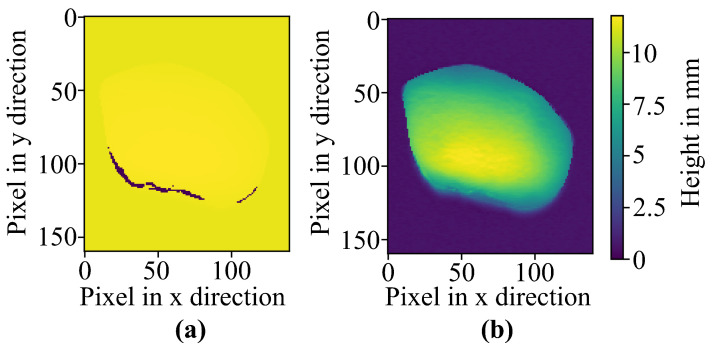
Results of stone one (object). (**a**) False color images of depth shadows imaged by the 3D laser line scanner (height legend: yellow > 0 mm, and purple = 0 mm) and the (**b**) improved depth map.

By using a 3D laser line scanner, the depth images are subjected to artifacts called depth shadows (along the edges), as seen in Figure 3. These artifacts are an inaccuracy, as can be seen from the depth value reading zero in the image, causing a bigger offset between the RGB image and depth data. Removing these artifacts is necessary to fuse the images accurately. With the help of interpolation and averaging over neighboring pixels, in addition to a threshold-based edge detection algorithm, the removal of the artifacts was achieved. Using this algorithm, the edges of the depth shadow were removed, and, therefore, the edges of the depth data were improved, resulting in sharper edges closer to the real edges of the imaged object.

## 4. Data Acquisition and Processing

**Figure 4:**
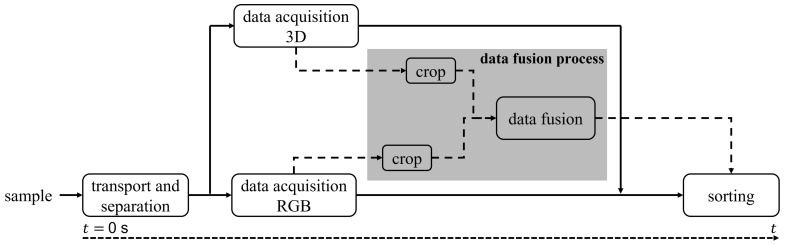
Flowchart of the data acquisition process.

**Figure 5:**
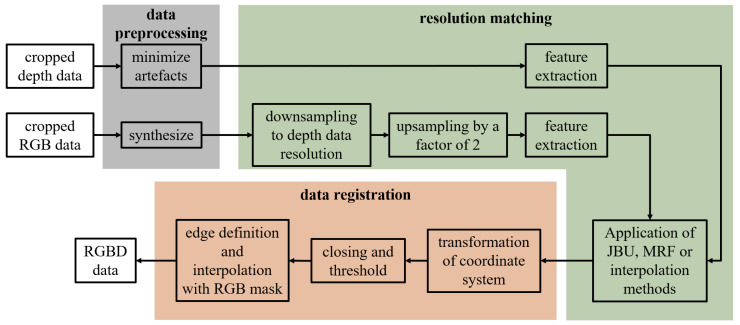
Flowchart of the data fusion process.

In order to understand the complete process, different levels of processing needed to be considered. First, one needs to understand what happens in data acquisition. This process is shown in Figure 4. In order to take a closer look at the data fusion process in the grey area in Figure 4, the flowchart in Figure 5 is shown.

Since the camera systems are positioned along the conveyor belt behind each other, there is a temporal difference, Δt, in acquisition. Since both data need to be present to combine them, the processing of both data needs to be delayed until both data are acquired. For segmentation, a cropping algorithm is used. This, however, can be carried out right after acquisition.

An overview of the data fusion process is shown in Figure 5. Before the preprocessing algorithm is used, the incoming samples of the conveyor belt are cropped to remove the background. Afterward, the preprocessing algorithm is used on the acquired samples. On the one hand, the RGB data need to be synthesized to create an image of the full object without the RGB artifacts caused by the conveyor belt. Meanwhile, the depth shadows mentioned in Figure 3 are removed from the 3D data.

Next, resolution matching is implemented. This step uses different up- and downsampling steps to increase the resolution of the depth data and fit the RGB data accordingly. First, a combination of down- and upsampling methods based on classical interpolation methods is applied to the RGB data to create a resolution difference between RGB and depth data of a factor of 2 × 2.

The downsampling is necessary due to the ROI issue, as described in Section 3. The resolution of both data is not exactly an integer multiple of each other. The RGB data were chosen as reference data due to their higher source resolution. So, the RGB data were first downsampled to the resolution of the depth data. In the second step, they are upsampled by a factor of two to meet the requirement for guided upsampling methods such as JBU and MRF.

Next, the depth data need to be assigned to their corresponding RGB data. This is carried out by feature extraction and feature matching. Features such as area, convexity, etc., are extracted from the data and assigned over difference tolerances. The features used are the following:

- Geometric dimensions: height, width, and area;
- Circumference;
- Center of gravity;
- Convexity;
- Upper-edge position.

After the assignment, the JBU, MRFs, and classical interpolation methods are finally applied, respectively, to match the RGB data resolution and the depth data resolution. Since the RGB data resolution was set to double the depth data resolution in the previous steps, the depth data were upsampled by a factor of 2 × 2. For classical interpolation methods, the RGB data are not used. However, since JBU and MRFs are guided upsampling methods, RGB data with double the resolution are necessary (see Section 2.2 and Section 2.3).

As a last step, data registration is carried out. First, the co-ordinate system of the RGB data and the 3D data must be transformed into the same co-ordinate system to ensure an offset-free fusion process. With the transformation of the co-ordinate system, the fusion itself can be carried out. After fusion, a morphological operation, more precisely, closing, and a threshold to minimize the created noise are executed. In order to obtain a fully overlapping fusion of depth data and RGB data, points outside of the matching edges have to be eliminated by applying an RGB contour mask. Pixels inside the edges have to be interpolated to extend to the boundaries.

## 5. Evaluation Process

In order to be able to compare the different methods used for upsampling in terms of their performance for data fusion, different evaluation methods were used. For evaluation, the RMSE, CORR, SNR, and UQI based on the work of Jagalingam et al. [51] and Naidu et al. [52] are used. In addition to these assessment parameters, an offset between the depth data and RGB data was used. *N* and *L* are the number of pixels in each spatial dimension, and *I* corresponds to the intensity of the pixels at positions *i* and *j* of the depth data or RGB data, respectively.

RMSE measures the quadratic error between the source images, the RGB and upsampled depth data, and the fused RGB-D image, with zero being the best possible score. Equation (6) [51,52] can be used to calculate the RMSE:

(6) $$\mathrm{RSME}=\sqrt{\frac{1}{N\;\cdot \;L}\;\cdot \;\sum_{i=1}^{N}\sum_{j=1}^{L}{\left(I_{\mathrm{binary},\mathrm{upsampled},\mathrm{depth}}\left(i,j\right)-I_{\mathrm{binary},\mathrm{RGB}}\left(i,j\right)\right)}^{2}}$$

where $I_{\mathrm{binary},\mathrm{RGB}}$ represents the intensity of the image acquired by the RGB line camera, and $I_{\mathrm{binary},\mathrm{upsampled},\mathrm{depth}}$ is the intensity calculated using the acquired depth data and the respective upsampling method. In order to measure the information similarities between the source images and the fused image SNR, which can be seen in Equation (7) [51,52], this equation can be used. The higher the score, the better the method has performed:

(7) $$\mathrm{SNR}=20\;\cdot \;\log_{10}\left(\frac{\sum_{i=1}^{N}\sum_{j=1}^{N}I_{\mathrm{binary},\mathrm{upsampled},\mathrm{depth}}{\left(i,j\right)}^{2}}{\sum_{i=1}^{N}\sum_{j=1}^{N}{\left(I_{\mathrm{binary},\mathrm{upsampled},\mathrm{depth}}\left(i,j\right)-I_{\mathrm{binary},\mathrm{RGB}}\left(i,j\right)\right)}^{2}}\right),$$

CORR is the correlation coefficient given in Equations (10) and (11) [51,52]. It is used as an evaluation method to measure the similarity of an RGB or depth image and an RGB-D image, with 1 being a perfect score:

(8) $$C_{\mathrm{depth},\mathrm{RGB}}=\sum_{i=1}^{N}\sum_{j=1}^{L}I_{\mathrm{binary},\mathrm{upsampled},\mathrm{depth}}\left(i,j\right)\;\cdot \;I_{\mathrm{binary},\mathrm{RGB}}\left(i,j\right),$$

(9) $$C_{\mathrm{depth}}=\sum_{i=1}^{N}\sum_{j=1}^{L}I_{\mathrm{binary},\mathrm{upsampled},\mathrm{depth}}{\left(i,j\right)}^{2},$$

(10) $$C_{\mathrm{RGB}}=\sum_{i=1}^{N}\sum_{j=1}^{L}I_{\mathrm{binary},\mathrm{RGB}}{\left(i,j\right)}^{2},$$

(11) $$\mathrm{CORR}=\frac{2\;\cdot \;C_{\mathrm{depth},\mathrm{RGB}}}{C_{\mathrm{depth}}\;\cdot \;C_{\mathrm{RGB}}}\;$$

UQI is the universal quality index given in Equation (12) [51,52]. It is used to describe the information brought into the fused image, with 1 being the best possible score.

(12) $$\mathrm{UQI}=\frac{4\;\cdot \;\sigma_{depth,RGB}\;\cdot \;\left(\mu_{depth}+\mu_{RGB}\right)}{\left(\sigma_{depth}^{2}+\sigma_{RGB}^{2}\right)\;\cdot \;\left(\mu_{depth}^{2}+\mu_{RGB}^{2}\right)}\;,\;\mathrm{with}\;$$

- μ being the mathematical expected value, respectively;
- σ being the standard deviation, respectively.

The offset, $\Delta {\mathrm{Off}}_{position}$, is measured to evaluate the overlap of the object maps between the source and fused images. Equation (13) gives the if statement to calculate the offset:

(13) $$\Delta {\mathrm{Off}}_{position}=\left\{\begin{array}{cc} +1 & \;\mathrm{if}\;edge_{RGB}\left(i,j\right)\neq edge_{depth}\left(i,j\right),\\ 0 & \;\mathrm{otherwise}. \end{array}\right.$$

These assessment parameters are used to compare and evaluate the upsampling methods presented in Section 6.

## 6. Results

In total, more than 225 data fusion experiments using 11 different objects (10 stones and the validation coin) and upsampling parameters were conducted. Table 2, Table 3 and Table 4 show the mean of the evaluation parameters for each of the upsampling methods presented in Section 2. Based on the results, Table 5 shows a comparison using the best-performing parameters. The mean was calculated considering all analyzed objects. Due to computational costs, the upsampling of the factors to 2 × 2 the resolution of the RGB data was analyzed.

For MRFs, 30 different parameter scenarios were conducted to find the best parameters. Table 2 shows the mean evaluation results of Markov random field upsampling using a cubic interpolation algorithm [46] since the performance of the MRFs using nearest neighbor and bi-linear interpolation did not perform as well. $\sigma_{c}$ or β were varied while the other parameters were fixed. The variation in the parameters was chosen arbitrarily. A total of 60 iterations were carried out; for the majority of the dataset, this quantity of iterations was sufficient, and the performance was saturated. When comparing the negative and positive values of $\sigma_{c}$, equal results were achieved. However, varying the sign of β does show an effect. Based on this, the best parameters were chosen for upsampling using MRFs. In the first step of the experiments, $\sigma_{c}=1$ was set while β was varied, with β=0.99 achieving the best performance. For β=0.99, $\sigma_{c}$ was varied in the second step of experiments. With this arrangement, $\sigma_{c}=\frac{17}{255}$ performs the best. A third iteration of experiments was conducted to find the best fitting β value for $\sigma_{c}=\frac{17}{255}$. While $\sigma_{c}=\frac{17}{255}$ and β=10 performed best, $\sigma_{c}=\frac{17}{255}$ and β=0.99 also performed well. Since these parameters are recommended by [46], choosing these parameters ensures comparability with other publications.

Table 3 displays the evaluation results for different up- and downsampling scenarios for each interpolation method. Direct downsampling and upsampling were achieved in one process without any intermediate sampling stages, whereas in other scenarios, the resolution was adjusted in multiple cascading processes. In each scenario, nearest neighbor and bi-linear interpolation outperform cubic interpolation. Moreover, the best result was achieved by iterating the downsampling process by a factor of 2 × 2 with nearest neighbor interpolation.

Table 4 gives an overview of the JBU performance for different upsampling scenarios. All evaluation parameters are the means and standard deviations of multiple objects. Overall, one can see JBU performs best with a nearest neighbor interpolation. Moreover, downsampling the RGB data by a factor of 4 × 4 and later upsampling the data to the depth data resolution results in the best performance in terms of the assessment parameters chosen in this work.

Based on two example objects, all the evaluation values are given in Table 5. For each upsampling method, the best-performing parameter setup is shown. For Markov random fields, n=60 iterations were used. $\Delta Off_{Position}$ is used in addition to RSME, CORR, and UQI to show the overlap of the RGB image and the depth map in the fused RGB-D image. Due to the cheap computational costs, JBU and interpolation fusion have an advantage over MRFs because of the iterations MRFs have to undergo in order to optimize the results. JBU clearly outperforms MRFs and interpolation in this work. Due to the guided fusion over a convolutional weight, JBU shows good performance in the fusion process. Nearest neighbor outperformes the bi-linear and cubic interpolation and is, therefore, more benefitial for preprocessing.

Naidu et al. [52] also achieved CORR=0.99. Moreover, the authors of [53] report correlation coefficients between 0.95 and 0.99 for various data fusion methods. Zhang [54] achieved a maximum of CORR=1 by modifying a multispectral image; however, other methods presented by [54] perfrom worse, with correlation coefficients between $CORR_{min}=0.72$ and $CORR_{max}=0.91$. When comparing the evaluation metrics RMSE, UQI, SNR, and CORR of JBU with Zhu et al. [53], Naidu et al. [52], and Zhang [54], we can see that JBU performance is similar and comparable. However, one must note that Zhu et al. [53] and Zheng [54] fused the spectral images, e.g., panchromatic, RGB, or multispectral images, whereas, in our work, the spatial and spectral data in the form of RGB and depth data were fused.

## 7. Conclusions

This publication compares different upsampling methods for their use in data fusion based on complex objects. Three different processes—classical interpolation methods, MRFs, and JBU—are compared. First, the best parameter for each method was determined before they were compared. By applying JBU, downsampling the RGB data by a factor of 4 × 4, and later upsampling the data to the depth data resolution, as well as using nearest neighbor interpolation, the other methods were outperformed.

**Figure 6:**
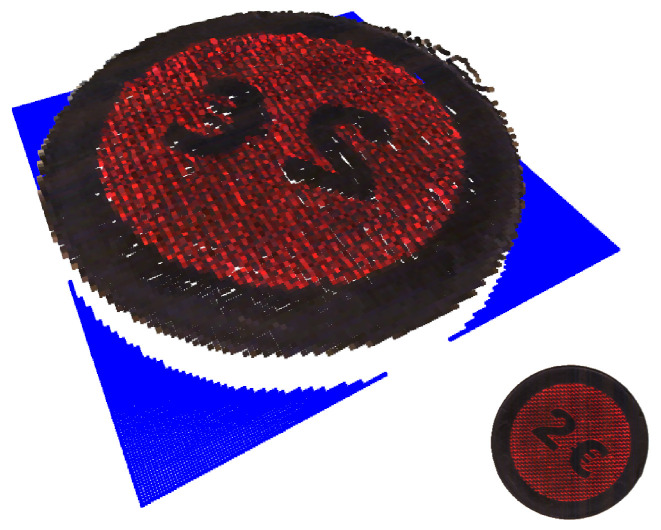
RGB-D point cloud of the validation coin fused by JBU.

Figure 6 shows the RGB-D point cloud of the validation coin. One can see the texture on the coin combined with the height profile, with a total diameter of 23 mm; the height resolution matches the texture resolution provided by the RGB image.

**Figure 7:**
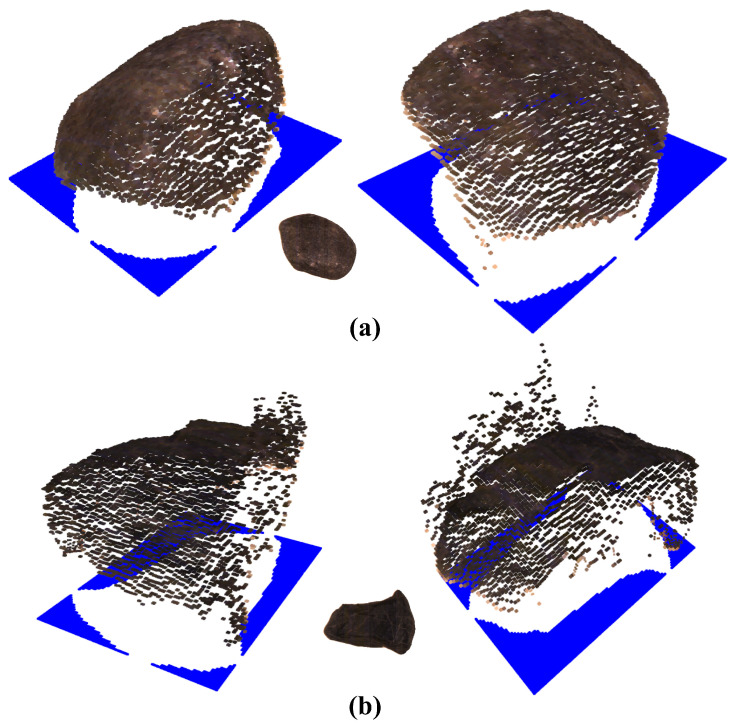
RGB-D point cloud after data fusion, for example, stones-A (**a**) and -B (**b**) fused by JBU.

Figure 7 shows the resulting RGB-D point cloud for the two example stones, which were retrieved using the described JBU setup. By using a 3D laser line scanner and an RGB line camera, high-resolution RGB-D images were created. This enables volume approximation and the detailed location of textual and form-based features. However, there are also flaws seen in Figure 7b. Figure 7b shows noisy depth points due to the rough or reflective topography of the analyzed object, which leads to measurement errors in the depth measurement due to the scattering of the laser beam. Smoothing over this region via the neighborhood would lead to fewer deviations.

**Figure 8:**
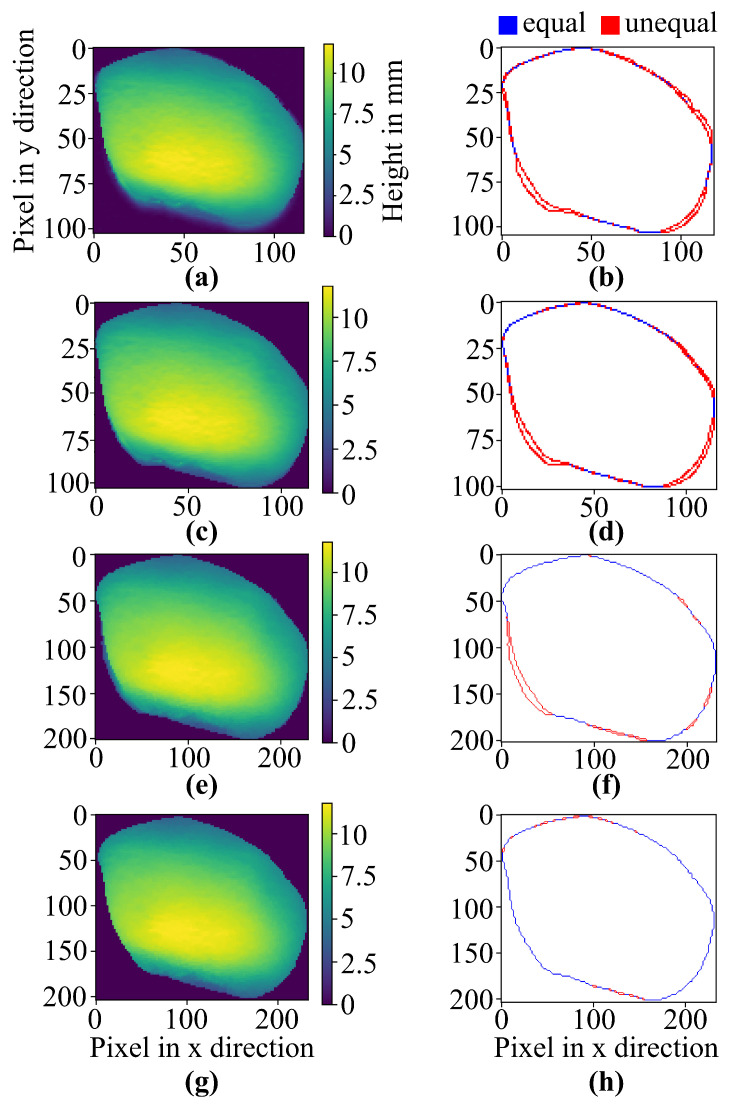
Results of object stone-A. False color image depth maps during the fusion process (**a**,**c**,**e**,**g**) and the edge correlation of the depth map and RGB image (**b**,**d**,**f**,**h**). (**a**,**b**) show the process result of the cropped depth data, with minimized depth shadows; (**c**,**d**) show the process result of the depth data after the synthesis; (**e**,**f**) show the process result after JBU data fusion; (**g**,**h**) show the process result after the feature extraction process.

Figure 8 shows the results of the different steps of data processing, for example, stone number one. Figure 8a shows the depth map of the cropped depth data, whereas Figure 8b shows the edge correlation between the RGB image and the depth map. After minimizing the artifacts of the depth shadow, a blurry edge at the bottom of Figure 8a can be seen. This noisy region is due to the depth shadows. Figure 8c shows the results of the synthesized depth map of Figure 8a. The synthesizing step uses a threshold defined by the histogram of the depth map in order to minimize these blurry edges. Figure 8d shows an improvement in correlation due to the exclusion of depth shadows. Figure 8e,f depict the result of the JBU data fusion process. Using JBU increases the correlation between the depth data and RGB data but leads to the synthesized RGB image having a few noise residuals, which are visible in the blurry bottom left part of Figure 8e. Feature extraction of the RGB image is used in order to create an edge mask to cut out these blurry residuals and overlap both images. With this step, the correlation of the edges increases even further, as depicted in Figure 8g,h. For the final interpolation of the missing depth map edges with the neighborhood of the missing data, the edge matching of the RGB data and depth data can be achieved.

To conclude this work, data fusion was used for image enhancement by removing artifacts and improving the depth map edge quality by removing the depth shadow and matching the edges of the RGB data and depth data. Moreover, different interpolation methods for RGB data and depth map fusion are compared for multiple assessment parameters. The parameters of each interpolation method were varied to find the best-performing settings. The results show JBU outperforming MRFs and the direct interpolation methods. This work shows the capabilities of RGB-D imaging with temporarily separated imaging systems. The setup-based time delay between both imaging systems was solved using feature extraction and data registration. By imaging RGB data and depth data separately along a conveyor belt system and fusing additional information, the quality of the imaged data was improved.

In future works, other data fusion methods will be compared, including the novel polygon-based approach of triangle-mesh-rasterization projection (TMRP) [41]. Moreover, the spectral dimension in this work is confined to RGB, and this will be expanded by using multispectral and hyperspectral imaging systems in addition to the RGB camera system.

## Figures and Tables

**Table 1:** Attributes for each imaging system used in the system [49,50].

Attribute	RGB Camera	3D Laser Line Scanner
	JAI CV-L107 CL	Scan CONTROL 3000-200
Imaging profile	RGB line camera	Lasertriangulation
No. of active pixels	3 × 2048	1 × 1024
Pixel size	14 μm × 14 μm	26 μ m× 26 μm

**Table 2:** Mean and standard deviation of MRFs with different parameters and cubic interpolatiosn for 60 iterations.

Parameter		RMSE	SNR	CORR	UQI
$\sigma_{c}=1$	β=0	53.545±9.16	16.99±4.05	0.92±0.04	0.91±0.03
	β=0.25	51.75±8.69	17.65±4.13	0.93±0.034	0.91±0.03
	β=0.5	51.01±8.42	17.92±4.11	0.93±0.03	0.91±0.03
	β=0.75	50.53±8.19	18.01±4.08	0.93±0.03	0.91±0.03
	β=1	137.00±20.63	nan	nan	0.67±0.1
	β=10	63.45±38.25	17.45±3.35	0.93±0.03	0.86±0.14
	β=2	51.12±7.51	15.80±6.79	0.82±0.29	0.91±0.02
	β=−1	86.94±27.20	10.20±3.74	0.79±0.13	0.83±0.08
	β=−10	57.72±9.66	15.49±2.78	0.91±0.03	0.90±0.03
	β=0.99	50.29±8.12	18.18±4.07	0.93±0.03	0.91±0.03
β=0.99	$\sigma_{c}=0.5$	48.62±4.91	18.68±3.63	0.94±0.03	0.92±0.02
	$\sigma_{c}=\pm \frac{17}{255}$	51.05±8.12	17.89±4.05	0.93±0.03	0.91±0.03
	$\sigma_{c}=\pm 1$	50.23±8.11	18.18±4.07	0.93±0.03	0.91±0.03
	$\sigma_{c}=\pm 10$	90.22±41.43	12.26±7.29	0.80±0.14	0.76±0.17
	$\sigma_{c}=0.25$	51.20±8.09	17.83±4.04	0.93±0.03	0.91±0.03
	$\sigma_{c}=\pm 40$	138.60±36.33	6.22±2.35	0.66±0.12	0.61±0.17
$\sigma_{c}=\frac{17}{255}$	β=0	53.54±9.16	16.99±4.05	0.92±0.04	0.91±0.03
	β=0.25	52.13±8.72	17.50±4.09	0.92±0.04	0.91±0.03
	β=0.5	51.59±8.44	17.70±4.07	0.93±0.03	0.91±0.03
	β=0.75	51.19±8.26	17.84±4.05	0.93±0.03	0.91±0.03
	β=1	137.00±20.63	nan	nan	0.67±0.10
	β=10	50.86±8.07	17.95±4.05	0.93±0.03	0.91±0.03
	β=2	50.90±8.13	17.94±4.05	0.93±0.03	0.91±0.03
	β=−1	137.00±20.63	nan	nan	0.67±0.10
	β=−10	51.05±8.11	17.87±4.13	0.93±0.03	0.91±0.03
	β=0.99	51.05±8.20	17.89±4.05	0.93±0.03	0.91±0.03

**Table 3:** Mean and standard deviation of different single-interpolation methods and parameters.

DS * and US * Scenario and Interpolation Method		RMSE	SNR	CORR	UQI
Direct DS*	nearest neighbor	46.81±8.39	18.98±3.86	0.94±0.02	0.91±0.031
	bi-linear	52.78±9.79	16.77±4.27	0.92±0.04	0.89±0.04
	cubic	53.19±9.29	16.60±4.00	0.92±0.04	0.89±0.04
Direct DS *-factor 2 × 2	nearest neighbor	47.33±9.97	18.71±3.94	0.94±0.03	0.89±0.05
	bi-linear	52.08±9.23	16.37±4.00	0.92±0.04	0.88±0.05
	cubic	59.99±7.36	12.32±2.96	0.89±0.03	0.85±0.04
DS *-factor 2 × 2	nearest neighbor	45.03±8.66	19.53±3.93	0.94±0.02	0.90±0.04
	bi-linear	50.98±10.88	16.86±4.58	0.92±0.04	0.87±0.06
	cubic	54.98±8.02	13.89±2.99	0.90±0.03	0.87±0.04
DS *-factor 4 × 4	nearest neighbor	47.85±11.86	19.00±4.92	0.93±0.04	0.88±0.06
	bi-linear	49.63±9.80	17.27±4.01	0.92±0.04	0.84±0.09
	cubic	52.93±8.64	14.57±2.58	0.91±0.02	0.86±0.06
Direct DS *-factor 4 × 4	nearest neighbor	51.20±12.04	17.78±4.38	0.93±0.04	0.86±0.07
	bi-linear	49.41±10.35	17.37±3.85	0.93±0.03	0.86±0.07
	cubic	57.73±8.83	13.02±2.95	0.89±0.03	0.82±0.05
Direct US *	nearest neighbor	55.32±9.73	15.98±4.13	0.91±0.04	0.91±0.03
	bi-linear	53.45±8.64	15.81±3.68	0.92±0.03	0.91±0.03
	cubic	64.94±7.54	10.92±2.80	0.87±0.04	0.89±0.02
US *-factor 2 × 2	nearest neighbor	55.32±9.73	15.98±4.13	nan	0.93±0.02
	bi-linear	52.50±8.64	15.82±3.68	nan	0.93±0.02
	cubic	69.42±7.67	8.78±2.84	nan	0.90±0.02

* DS: Downsampling to the resolution of the depth data; US: Upsampling to the resolution of the RGB data.

**Table 4:** Mean and standard deviation of JBU with different interpolation methods and parameters.

Variant		RMSE	SNR	CORR	UQI
Direct US *	nearest neighbor	40.93±9.07	21.49±4.89	0.95±0.02	0.94±0.02
	bi-linear	71.59±8.81	12.43±3.10	0.86±0.05	0.76±0.06
	cubic	69.48±7.83	12.91±3.10	0.87±0.05	0.69±0.08
DS * and US *-factor 2 × 2	nearest neighbor	38.35±10.60	23.03±5.37	0.96±0.03	0.93±0.05
	bi-linear	83.26±9.81	8.96±3.87	0.75±0.12	0.63±0.12
	cubic	84.59±10.52	8.72±3.74	0.74±0.12	0.55±0.16
DS * and US *-factor 4 × 4	nearest neighbor	25.22±3.44	32.80±9.62	0.99±0.02	0.97±0.05
	bi-linear	118.61±26.90	10.28±6.62	0.74±0.17	0.55±0.29
	cubic	124.63±22.01	9.25±5.42	0.73±0.15	0.53±0.27
Procedure similar to that in [21]	nearest neighbor	33.17±9.19	25.68±6.05	0.97±0.02	0.96±0.02
	bi-linear	89.72±8.27	9.05±2.24	0.77±0.07	0.77±0.05
	cubic	85.15±8.31	9.74±2.40	0.79±0.06	0.69±0.06
US *-factor 4 × 4 similar as in [21]	nearest neighbor	44.18±9.26	20.15±4.62	nan	0.96±0.02
	bi-linear	62.32±8.84	14.46±3.55	nan	0.91±0.03
	cubic	57.22±9.22	15.86±3.83	nan	0.90±0.03

DS *: Downsampling to the resolution of the depth data; US *: Upsampling to the resolution of the depth data.

**Table 5:** Overview of the presented upsampling method performances for two example stones.

Example Stone	RMSE	SNR	CORR	UQI	$\Delta {\mathrm{Off}}_{\mathrm{position}}$
Stone-A					
Interpolation NN	41.00	19.67	0.95	0.92	4.69%
MRF (n * = 60)	43.23	19.55	0.95	0.93	2.86%
JBU	11.40	42.46	1.00	0.99	0.25%
Stone-B					
Interpolation NN	61.29	14.75	0.91	0.84	8.83%
MRF (n * = 60)	53.26	18.28	0.94	0.90	3.99%
JBU	11.64	44.60	1.00	0.99	0.24%

* n: number of iterations; NN: nearest neighbor.

## Data Availability

The data and software that support the findings of this study are available from the corresponding author, [L.W.], upon request.
